# Serum bicarbonate is a marker of peri-operative mortality but is not associated with long term survival in colorectal cancer

**DOI:** 10.1371/journal.pone.0228466

**Published:** 2020-01-30

**Authors:** Joseph Chung Yan Chan, Connie Irene Diakos, Alexander Engel, David Lok Hang Chan, Nick Pavlakis, Anthony Gill, Stephen John Clarke

**Affiliations:** 1 Bill Walsh Translational Research Laboratories, Kolling Institute of Medical Research, St Leonards, New South Wales, Australia; 2 Sydney Medical School, University of Sydney, Sydney, New South Wales, Australia; 3 Northern Sydney Cancer Center, Royal North Shore Hospital, St Leonards, New South Wales, Australia; 4 Department of Colorectal Surgery, Royal North Shore Hospital, St Leonards, New South Wales, Australia; 5 Cancer Diagnosis and Pathology Group, Kolling Institute of Medical Research, St Leonards, New South Wales, Australia; University Hospital Hamburg Eppendorf, GERMANY

## Abstract

**Aims:**

Inflammation is a hallmark of cancer whose activity is modulated within the tumor microenvironment by low tumoral pH. Recent evidence in the literature has suggested a link between low serum bicarbonate, low tumoral pH and cancer related inflammation. There is however little clinical evidence in human patients regarding the prognostic role of serum bicarbonate. Therefore, the primary aim of this study was to investigate the short and long-term prognostic utility of serum bicarbonate in colorectal cancer (CRC) patients undergoing resection of their primary tumor. The study also aimed to investigate the association of serum bicarbonate with known markers of systemic inflammation.

**Methods:**

A total of 3281 consecutive patients who underwent surgical resection of their primary CRC from January 1998 to December 2012. Of these, 2223 stage I-IV patients had available data for analysis. The association of serum bicarbonate with overall survival was assessed using univariate and multivariate cox regression analyses. The association of bicarbonate with other clinicopathological variables was assessed by chi squared and Fisher’s exact tests.

**Results:**

Serum bicarbonate was associated with peri-operative mortality in multivariate analysis (p<0.001). Age (p = 0.004), grade (p = 0.043), creatinine (p = 0.036) and sodium (p = 0.036) were also markers associated with peri-operative mortality. For long term survival at 5 years, bicarbonate was significantly associated with overall survival in univariate analysis (p<0.001) but was not significant in multivariate analysis (p = 0.075). In exploratory analysis, serum bicarbonate was found to be significantly associated with the lymphocyte-to-monocyte ratio (p<0.001) and neutrophil-to-lymphocyte ratio (p<0.001).

**Conclusions:**

In peri-operative colorectal cancer patients, serum bicarbonate was associated with 30-day survival but not 5-year survival.

## Introduction

Cancer related inflammation is widely recognized as an emerging hallmark of cancer and its role in initiating and aiding in the progression of cancer has been well studied [1]. Inflammation, particularly when chronic, can facilitate processes that lead to genomic instability and carcinogenesis [2]. This process has been well described in patients with inflammatory bowel disease whose risk of developing colorectal cancer (CRC) is directly correlated with the duration of their disease [3].

In established malignancy, inflammation both systemically and in the tumor microenvironment plays a role in modulating cancer activity[4]. The balance between players that drive anti-tumorigenic and pro-tumorigenic activity is influenced by key factors such as tumoral pH. There is now evidence that an acidic tumor microenvironment is associated with inflammatory processes that are primarily pro-tumorigenic[5]. This includes inhibition of tumor infiltrating lymphocyte (TIL) cytotoxic activity and also polarizing tumor associated macrophages (TAMs) to the pro-tumorigenic M2 phenotype [6].

The link between tumoral pH and pro-tumorigenic inflammation has driven substantial interest recently in developing and utilizing therapeutic interventions aimed at manipulating tumoral pH [7]. This has been further fueled by evidence in mouse models that oral supplementation can effectively raise tumoral pH and reduce rates of spontaneous metastasis [8]. However, there is no evidence currently that alkalinizing diets, treatments or oral bicarbonate are effective anti-cancer treatments in human subjects [9]. Moreover, the prognostic implications of a low baseline serum bicarbonate in a cancer cohort remain completely undefined within the literature. Whether there is even a theoretical basis for altering serum bicarbonate in a human cancer cohort remains unclear. Certainly, this lack of data regarding bicarbonate levels in cancer patients has allowed alkalinizing treatments to remain in an ambiguous therapeutic space. Defining the relationship of serum bicarbonate levels with systemic inflammation and survival in a cancer cohort will be invaluable in clarifying any potential therapeutic role of bicarbonate in human patients.

Thus, the principal aim of the current study was to investigate the peri-operative and longer-term prognostic utility of baseline serum bicarbonate in colorectal cancer (CRC) patients undergoing resection of their primary tumors. Our secondary aims was to investigate the association of serum bicarbonate with established markers of systemic inflammation.

## Methods and materials

### Patient cohort

The total cohort consisted of 3281 consecutive patients from the Northern Sydney Local Health District in Sydney, Australia who underwent primary surgical resection of their CRC. These patients, who presented for surgical resection between January 1998 and December 2012, underwent operations at six hospitals including both quaternary and community hospitals with surgical units. Some data such as gender, age and grading were prospectively collected. All samples were reviewed and staged according to the AJCC 7^th^ edition 2009 staging system [10].

Patients in the cohort received adjuvant treatment according to accepted guidelines based on stage. For example, standard adjuvant chemotherapy was offered to suitable patients with stage III and high risk stage II colon cancer. In comparison, neoadjuvant chemoradiotherapy was routinely offered to patients with stage II/III rectal cancers. Stage IV CRC patients in the cohort were only those who had undergone resection of their primary tumors. Patients undergoing systemic treatments or palliative management without operations were excluded. Patients underwent treatment at several facilities including both public and private treatment centres. Complete data regarding chemotherapy dosing, toxicity, time of relapse and subsequent therapy received for metastatic disease were not available.

The follow up of patients differed across centres and were based on individual physician preferences. Generally, patients were followed up every 3–6 months. Follow up usually included regular (3–6 monthly for 5 years) full clinical history, physical examination, a panel of bloods and a yearly computed tomography (CT) of chest, abdomen and pelvis [11].

### Immunohistochemistry

Immunohistochemical methods previously validated to be both highly sensitive and specific were used to evaluate resected tumour specimens for BRAFV600E mutation and mismatch repair (MMR) status [12, 13].

### Blood tests

Blood tests for patients varied based on physician preference but usually included a full blood count (FBC) and serum biochemistry. For the current study, the most recent blood tests in the 30 days prior to surgery was utilized for analysis. This data was collected retrospectively.

### Survival data

The primary endpoint of the current study was overall survival (OS), which was measured from the date of surgery until date of last follow up or the date of death from any cause. Follow up data on survival and death were collected from hospital pathology databases, private offices, electronic medical records, central death registries and publicly available death records up to November 2015.

### Statistics

The low and high groups for the lymphocyte-to-monocyte ratio (LMR) and neutrophil-to-lymphocyte ratio (NLR) were constructed by taking the ratio of the absolute count of the respective component of the full blood count. Patient data were dichotomized into ‘low’ and ‘high’ groups using a cutpoint of 2.38 and 3.75 for LMR and NLR respectively. These cutpoints were determined using the R package Maxstat which determined the cutpoint that was associated with the greatest log rank statistic between groups [14]. Cutpoints for other serum tests, where applicable, were identical to those used in the prior POSSUM score. If a serum test was not part of the POSSUM score, then laboratory reference ranges as determined by the set of values that 95% of the normal population fall within, were used.

The association of bicarbonate and clinicopathological variables were assessed using a chi squared test or Fisher’s exact test. The association between clinicopathological variables and overall survival evaluated using the log-rank test and represented by Kaplan Meier curves. Variables found to be significant in univariate analysis (p<0.05) were then included in multivariate analysis using a backwards Cox proportional hazards model.

The quality of this study was reported using the REMARK checklist (S1 Table). For all statistical tests a p value of < 0.05 was considered significant. Analyses were performed using SPSS software version 25 (SPSS Inc., Chicago, IL, USA) and R (Version 3.5.2). This study was approved by the NSLHD Human Research Ethics Committee under protocol 1201-035M and RESP/14/97. Patient data was not fully anonymized during initial collection of data into a database. However, all patient data were fully anonymized in subsequent access and analysis.

## Results

### Baseline characteristics

**Fig 1:**
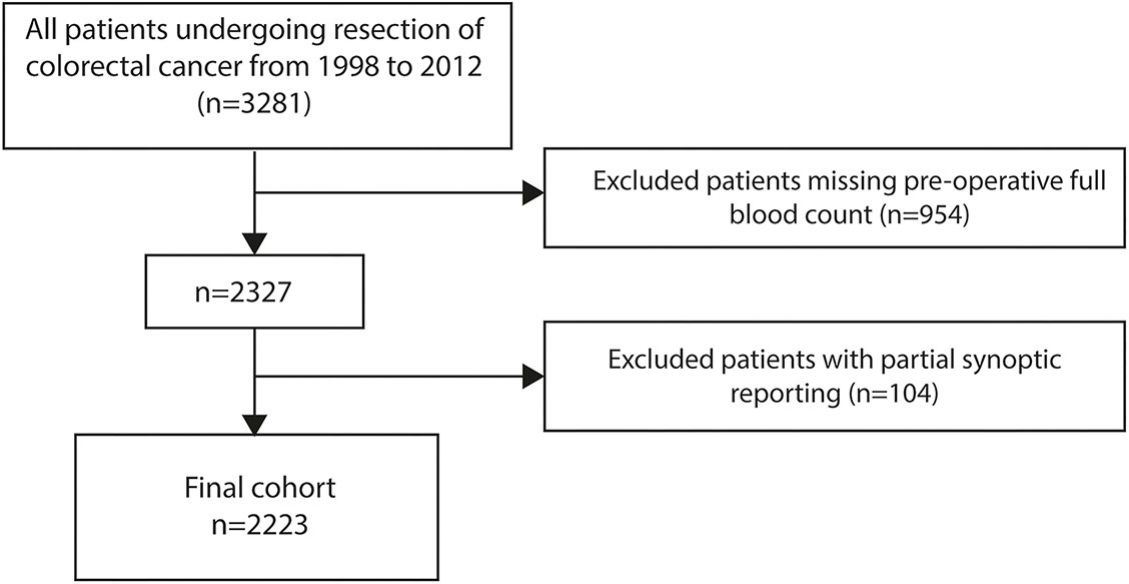
Flow chart of patient selection based on inclusion and exclusion criteria.

Of the 3281 patients in the cohort, 954 patients were excluded for not having blood tests in the 30 days prior to resection. A further 104 patients were excluded for having partial synoptic reporting. A total of 2223 patients with appropriate data were available for analysis (Fig 1). In brief, the cohort consisted of approximately equal number of males (49.7%) and females (50.3%). Patients were also more frequently older than 70 (60.1%). Most patients were T stage 3 (52.8%) or 4 (25.2%) and N stage 0 (53.7%). The full breakdown of baseline characteristics can be seen in Table 1.

**Table 1:** Baseline characteristics of 2223 patients with available blood test data.

Clinicopathologic variables	No., (%),	Low HCO3 (<22) n = 263	Normal HCO3 (> = 27) n = 1585	High HCO3 (> = 30) n = 361	P
**Gender**					
M	1104 (49.7)	128 (48.5)	782 (49.0)	194 (53.3)	0.332
F	1119 (50.3)	136 (51.5)	813 (51.0)	170 (46.7)	
**Age**					
≤70	887 (39.9)	76 (28.8)	650 (40.8)	161 (44.2)	<0.001
>70	1336 (60.1)	188 (71.2)	945 (59.2)	203 (55.8)	
**T stage**					
1	140 (6.3)	8 (3.0)	91 (5.7)	41 (11.3)	<0.001
2	349 (15.7)	30 (11.4)	242 (15.2)	77 (21.2)	
3	1174 (52.8)	130 (49.2)	865 (54.2)	179 (49.2)	
4	560 (25.2)	96 (36.4)	397 (24.9)	67 (18.4)	
**N stage**					
0	1193 (53.7)	130 (49.2)	844 (52.9)	219 (60.2)	0.056
1	657 (29.6)	89 (33.8)	478 (30.0)	90 (24.7)	
2	373 (16.8)	45 (17.0)	273 (17.1)	55 (15.1)	
**M stage**					
0	2123 (95.5)	10 (3.8)	81 (5.1)	9 (2.5)	0.052
1	100 (4.5)	254 (96.2)	1514 (94.9)	355 (97.5)	
**Site**					
Right-sided colon	1043 (46.9)	132 (50.0)	771 (48.3)	140 (38.5)	<0.001
Left-sided colon	611 (27.5)	83 (31.4)	427 (26.8)	101 (27.7)	
Rectum	569 (25.6)	49 (18.6)	397 (24.9)	123 (33.8)	
Grade*					
Low	919 (45.8)	71 (29.3)	635 (69.1)	213 (65.0)	<0.001
Mod	698 (34.8)	115 (47.5)	519 (74.8)	64 (19.5)	
High	389 (19.4)	56 (23.2)	282 (74.4)	51 (15.5)	
**MMR-BRAF status**†					
MMRp/BRAFV600E	175 (9.3)	20 (8.8)	131 (9.7)	24 (8.0)	0.076
MMRd/BRAFwt	96 (5.1)	7 (3.1)	79 (5.9)	10 (3.3)	
MMRd/BRAFV600E	215 (11.5)	28 (12.4)	162 (12.0)	25 (8.3)	
MMRp/BRAFwt	1388 (74.1)	171 (75.7)	975 (72.4)	242 (80.4)	
**WBC (x10**^**9**^ **cells /L)**					
4–10	1697 (76.3)	154 (58.3)	1227 (77.0)	316 (86.8)	<0.001
10.1–20 or 3.1–4	484 (21.8)	94 (35.6)	345 (21.6)	45 (12.4)	
>20 or < 3	42 (1.9)	16 (6.1)	23 (1.4)	3 (0.8)	
**Lymphocyte to monocyte ratio (LMR)**					
Low (<2.38)	1148 (51.6)	175 (66.3)	819 (51.3)	154 (42.3)	<0.001
High (> = 2.38)	1075 (48.4)	89 (33.7)	776 (48.7)	210 (57.7)	
**Neutrophil to lymphocyte ratio (NLR)**					
Low (<3.75)	1143 (51.4)	66 (25.0)	840 (52.7)	237 (65.1)	<0.001
High (> = 3.75)	1080 (48.6)	198 (75.0)	755 (47.3)	127 (34.9)	
**Creatinine** (mmol/L) ‡					
Low < 45/60	86 (3.8)	24 (9.1)	54 (3.4)	8 (2.2)	<0.001
Mod 45–90 / 60–110	1791 (80.6)	171 (64.8)	1312 (82.2)	308 (84.6)	
High >90 / 110	346 (15.6)	69 (26.1)	229 (14.4)	48 (13.2)	
**Haemoglobin (mg/dL)**					
13-16g/ dl	783 (35.2)	68 (25.8)	526 (33.0)	189 (52.0)	<0.001
11.5–12.9 or 16.1–17	576 (25.9)	59 (22.3)	435 (27.3)	82 (22.5)	
10–11.4 or 17.1–18	528 (23.8)	86 (32.6)	383 (24.0)	59 (16.2)	
<10 or > 18	336 (15.1)	51 (19.3)	251 (15.7)	34 (9.3)	
**Urea (**mg/dL)					
<7.6	1823 (82.0)	197 (74.6)	1308 (82.0)	318 (87.4)	<0.001
7.6–10.0	244 (11.0)	29 (11.0)	181 (11.3)	34 (9.3)	
10.1–15	125 (5.6)	22 (8.3)	91 (5.7)	12 (3.3)	
>15	31 (1.4)	16 (6.1)	15 (0.9)	0 (0.0)	
**Sodium (**mEq/L)					
>135	1937 (87.1)	228 (86.4)	1377 (86.3)	332 (91.2)	0.001
131–135	248 (11.2)	24 (9.1)	195 (12.2)	29 (8.0)	
126–130	34 (1.5)	11 (4.2)	20 (1.3)	3 (0.8)	
<126	4 (0.2)	1 (0.4)	3 (0.2)	0 (0.0)	
**Potassium (mEq/L)**					
3.5–5.0	2045 (9.2)	236 (89.4)	1480 (92.8)	329 (90.4)	0.087
3.2–3.4 or 5.1.-5.3	136 (6.1)	18 (6.8)	90 (5.6)	28 (7.7)	
2.9–3.1 or 5.4–5.9	40 (1.8)	10 (3.8)	24 (1.5)	6 (1.6)	
<2.9 or >5.9	2 (0.1)	0 (0.0)	1 (0.1)	2 (0.3)	

* 133 patients missing tumor grade,

^†^349 patients missing MMR-BRAF status,

^‡^Creatinine values are lower for females

The median follow-up was 50 months with an interquartile range of 26–93 months. In total there were 767 deaths from any cause in the cohort.

### Association of bicarbonate with clinicopathological factors

A low bicarbonate was associated with older age (p < 0.001), more advanced T stage (p <0.001), tumor site (p<0.001), higher grade (p<0.001), white blood cell count (p<0.001), lower LMR (p<0.001), higher NLR (p<0.001), creatinine (p<0.001), haemoglobin (p<0.001), urea (p<0.001), sodium (p = 0.001) (Table 1).

### Peri-operative mortality

In the analysis of 30-day mortality in 2223 patients, age (p<0.001), grade (0.009), creatinine (p = 0.001), white blood cell count (p = 0.002), NLR (p = 0.040), urea (p<0.001), sodium (p = 0.006) and bicarbonate (p<0.001) were all significant variables in univariable analysis (Table 2). Gender, T stage, N stage, M stage, tumor site, MMR-BRAF status, hemoglobin, LMR and potassium were not significant in univariable analysis. In further multivariable analysis, only age (p = 0.004), grade (p = 0.043) and bicarbonate (p<0.001), creatinine (p = 0.036) and sodium (p = 0.036) were significant (Table 2).

**Table 2:** Primary univariate and multivariate analysis of key serum, inflammatory and genomic markers in relation to 30-day overall survival in CRC patients undergoing resection of their primary tumor.

Clinicopathologic variables	No., (%),	Univariate analysis, HR (95% CI)	P	Multivariate analysis, HR (95% CI)	P
**Gender**					
M	1104 (49.7)	1 (referent)	0.783		
F	1119 (50.3)	1.089 (0.594–1.995)			
**Age**					
≤70	887 (39.9)	1 (referent)	<0.001	1 (referent)	0.004
>70	1336 (60.1)	13.49 (3.260–55.81)		8.497 (2.020–35.75)	
**T stage**					
1	140 (6.3)	1 (referent)	0.075		0.375
2	349 (15.7)	0.199 (0.018–2.199)			
3	1174 (52.8)	1.320 (0.310–5.613)			
4	560 (25.2)	2.141 (0.495–9.265)			
**N stage**					
0	1193 (53.7)	1 (referent)	0.923		
1	657 (29.6)	0.980 (0.485–1.981)			
2	373 (16.8)	1.161 (0.517–2.607)			
**M stage**					
0	2123 (95.5)	1 (referent)	0.398		
1	100 (4.5)	1.659 (0.513–5.370)			
**Site**					0.714
Right-sided colon	1043 (46.9)	1 (referent)	0.113		
Left-sided colon	611 (27.5)	0.648 (0.313–1.345)			
Rectum	569 (25.6)	0.413 (0.170–1.004)			
Grade*					
Low	919 (45.8)	1 (referent)	0.009	1 (referent)	0.043
Mod	698 (34.8)	2.841 (1.226–6.583)		2.218 (0.923–5.329)	
High	389 (19.4)	3.878 (1.607–9.356)		3.153 (1.282–7.757)	
**MMR-BRAF status** †					
MMRp/BRAFV600E	175 (9.3)	1 (referent)	0.671		
MMRd/BRAFwt	96 (5.1)	0.731 (0.142–3.765)			
MMRd/BRAFV600E	215 (11.5)	0.962 (0.294–3.152)			
MMRp/BRAFwt	1388 (74.1)	0.624 (0.239–1.631)			
**Creatinine (**mmol/L) ‡					
Low < 45/60	1791 (80.6)	1.714 (0.405–7.254)	0.001	1.417 (0.325–6.188)	0.036
Normal 45–90 / 60–110	86 (3.9)	1 (referent)		1 (referent)	
High >90 / 110	346 (15.5)	3.516 (1.868–6.620)		2.464 (1.242–4.889)	
**WBC (x10**^**9**^ **cells /L)**					
4–10	1697 (76.3)	1 (referent)	0.002		0.383
10.1–20 or 3.1–4	484 (21.8)	1.417 (0.703–2.856)			
>20 or < 3	42 (1.9)	6.697 (2.343–19.142)			
**Lymphocyte to monocyte ratio (LMR)**					
Low (<2.38)	1148 (51.6)	1 (referent)	0.298		
High (> = 2.38)	1075 (48.4)	0.721 (0.389–1.335)			
**Neutrophil to lymphocyte ratio (NLR)**					
Low (<3.75)	1143 (51.4)	1 (referent)	0.040		0.964
High (> = 3.75)	1080 (48.6)	1.936 (1.030–3.639)			
**Haemoglobin (mg/dL)**					
13-16g/ dl	783 (35.2)	1 (referent)	0.111		0.222
11.5–12.9 or 16.1–17	576 (25.9)	2.739 (1.105–6.786)			
10–11.4 or 17.1–18	528 (23.8)	2.825 (1.127–7.081)			
<10 or > 18	336 (15.1)	2.684 (0.973–7.401)			
**Urea (**mg/dL)					
<7.6	1823 (82.1)	1 (referent)	<0.001		0.338
7.6–10.0	244 (10.9)	1.423 (0.547–3.707)			
10.1–15	125 (5.6)	4.092 (1.776–9.428)			
>15	31 (1.4)	9.847 (3.436–28.22)			
**Sodium (**mEq/L)					
>135	1937 (87.1)	1 (referent)	0.006	1 (referent)	0.036
131–135	248 (11.2)	2.053 (0.944–4.465)		2.042 (0.920–4.534)	
126–130	34 (1.5)	3.753 (0.898–15.68)		1.731 (0.405–7.394)	
<126	4 (0.2)	15.94 (2.175–116.8)		12.83 (1.654–99.52)	
**Potassium (mEq/L)**					
3.5–5.0	2045 (92.0)	1 (referent)	0.397		
3.2–3.4 or 5.1.-5.3	136 (6.1)	1.670 (0.595–4.693)			
2.9–3.1 or 5.4–5.9	40 (1.8)	2.945 (0.709–12.231)			
<2.9 or >5.9	2 (0.1)	0.01 (na)			
**Bicarbonate (**mEq/L)				5.983 (3.006–11.906)	<0.001
Low (< = 22)	1595 (71.7)	7.535 (4.015–14.14)	<0.001	1 (referent)	
Normal (23–29)	264 (11.9)	1 (referent)		1.192 (0.341–4.163)	
High (> = 30)	365 (16.4)	0.719 (0.212–2.439)		1.192 (0.341–4.163)	

* 133 patients missing tumor grade,

^†^349 patients missing MMR-BRAF status,

^‡^Female patients with creatinine have lower cutpoints than males

### Long-term overall survival

In the analysis of 5-year long term survival of 2223 patients, gender (p = 0.013), age (p<0.001), T stage (p<0.001), N stage (p<0.001), M stage (p<0.001), grade (p<0.001), MMR-BRAF status (p<0.001), Lymphocyte-to-monocyte ratio (p<0.001), Neutrophil-to-lymphocyte ratio (p<0.001), creatinine (p<0.001), urea (p<0.001) and bicarbonate (p<0.001) were significant variables in univariable analysis (Table 3). Tumor site was not significant. In multivariable analysis, age (p<0.001), T stage (p<0.001), N stage (p<0.001), M stage (p<0.001), grade (p = 0.002), MMR-BRAF status (p = 0.006), LMR (p<0.001), creatinine (p = 0.002) and urea (p = 0.002) remained significant prognostic variables. Bicarbonate was not significantly associated with OS (p = 0.075) (Table 3).

**Table 3:** Primary univariate and multivariate analysis of key serum, inflammatory and genomic markers in relation to 5-year overall survival in CRC patients undergoing resection of their primary tumor.

Clinicopathologic variables	No., (%),	Univariate analysis, HR (95% CI)	P	Multivariate analysis, HR (95% CI)	P
**Gender**					
M	1104 (49.7)	1 (referent)	0.013		0.077
F	1119 (50.3)	0.819 (0.700–0.959)			
**Age**					
≤70	887 (39.9)	1 (referent)	<0.001	1 (referent)	<0.001
>70	1336 (60.1)	1.805 (1.521–2.142)		1.633 (1.334–2.000)	
**T stage**					
1	140 (6.3)	1 (referent)	<0.001	1 (referent)	<0.001
2	349 (15.7)	0.719 (0.419–1.232)		0.660 (0.355–1.229)	
3	1174 (52.8)	1.988 (1.265–3.126)		1.402 (0.825–2.381)	
4	560 (25.2)	5.383 (3.413–8.491)		2.835 (1.647–4.882)	
**N stage**					
0	1193 (53.7)	1 (referent)	<0.001	1 (referent)	<0.001
1	657 (29.6)	1.798 (1.491–2.168)		1.281 (1.025–1.601)	
2	373 (16.8)	3.728 (3.067–4.532)		2.835 (1.647–4.882)	
**M stage**					
0	2123 (95.5)	1 (referent)	<0.001	1 (referent)	<0.001
1	100 (4.5)	4.221 (3.234–5.509)		2.478 (1.771–3.468)	
**Site**					
Right-sided colon	1043 (46.9)	1 (referent)			
Left-sided colon	611 (27.5)	1.025 (0.851–1.234)	0.218		
Rectum	569 (25.6)	0.859 (0.706–1.046)			
Grade*					
Low	919 (45.8)	1 (referent)	<0.001	1 (referent)	<0.001
Mod	698 (34.8)	1.306 (1.073–1.589)		1.381 (1.106–1.724)	
High	389 (19.4)	2.037 (1.640–2.529)		1.621 (1.248–2.105)	
**MMR-BRAF status** †					
MMRp/BRAFV600E	175 (9.3)	1 (referent)	<0.001	1 (referent)	0.010
MMRd/BRAFwt	96 (5.1)	0.432 (0.270–0.691)		0.778 (0.470–1.286)	
MMRd/BRAFV600E	215 (11.5)	0.433 (0.304–0.618)		0.501 (0.335–0.749)	
MMRp/BRAFwt	1388 (74.1)	0.545 (0.424–0.701)		0.786 (0.592–1.043)	
**Lymphocyte to monocyte ratio (LMR)**					
Low (<2.38)	1148 (51.6)	1 (referent)	<0.001	1 (referent)	<0.001
High (> = 2.38)	1075 (48.4)	0.490 (0.416–0.577)		0.637 (0.524–0.776)	
**Neutrophil to lymphocyte ratio (NLR)**					
Low (<3.75)	1143 (51.4)	1 (referent)	<0.001		0.422
High (> = 3.75)	1080 (48.6)	1.918 (1.634–2.251)			
**Bicarbonate** (mEq/L)					
Low (< = 22)	264 (11.9)	1.912 (1.545–2.365)	<0.001	1.277 (0.996–1.638)	0.075
Normal (23–29)	1595 (71.7)	1 (referent)		1 (referent)	
High (> = 30)	364 (16.4)	0.885 (0.705–1.111)		1.221 (0.934–1.597)	
**Creatinine (**mmol/L) ‡					
Low < 45/60	1791 (80.6)	2.061 (1.454–2.922)	<0.001	1.990 (1.320–3.001)	0.002
Normal 45–90 / 60–110	86 (3.9)	1 (referent)		1 (referent)	
High >90 / 110	346 (15.5)	1.730 (1.423–2.104)		1.286 (0.967–1.710)	
**Urea (**mg/dL)					
<7.6	1823 (82.1)	1 (referent)	<0.001	1 (referent)	0.002
7.6–10.0	244 (10.9)	1.329 (1.047–1.686)		1.074 (0.783–1.472)	
10.1–15	125 (5.6)	1.980 (1.490–2.630)		1.601 (1.110–2.307)	
>15	31 (1.4)	3.209 (1.949–5.284)		2.785 (1.520–5.101)	

* 133 patients missing tumor grade,

^†^349 patients missing MMR-BRAF status,

^‡^Female patients with creatinine have lower cutpoints than males

### Performance of serum bicarbonate as a predictor of mortality

**Fig 2:**
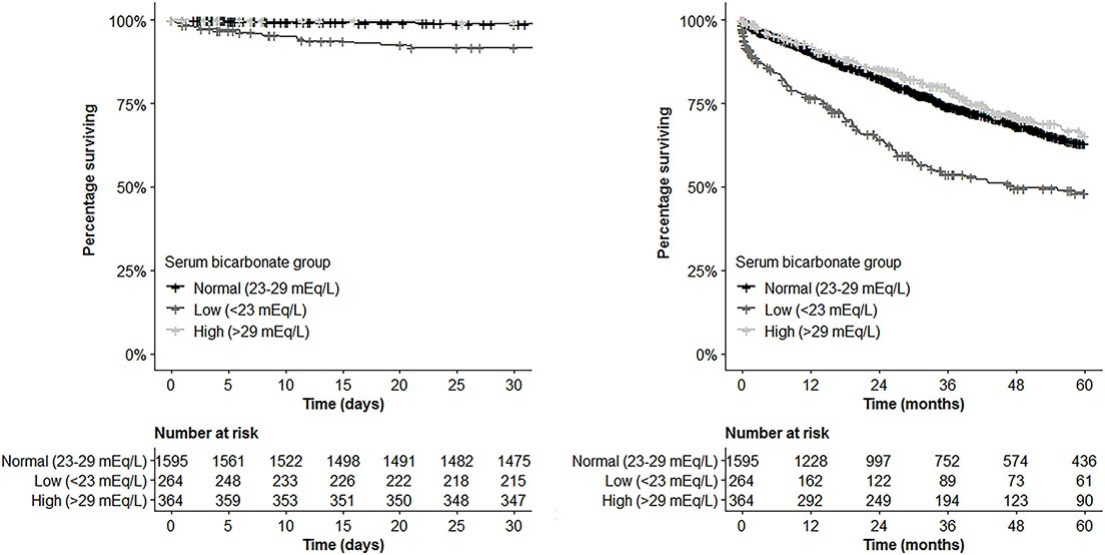
ROC curves for serum bicarbonate (Left) ROC curve for serum bicarbonate prediction of 30-day mortality (Right) ROC curve for serum bicarbonate prediction of 5-year overall survival.

ROC curve analysis was performed to assess the sensitivity and specificity of serum bicarbonate as a predictor of mortality. For 30-day mortality, tthe AUC was 0.752 (95% CI 0.671–0.834, p<0.001) (Fig 2). At the cut-off point of 22 mEq/L the sensitivity was 92.3% and the specificity was 31.8%. The optimal cutoff point was determined to be 25.0 mEq/L where the sensitivity was 75.4% and the specificity was 66.0%. For 5-year overall survival, the AUC was 0.559 (95% CI 0.532–0.586, p<0.001) (Fig 2). At the cut-off of 22 mEq/L the sensitivity was 93.9%. and specificity 11.2%. The optimal cutoff point was determined to be 27.0 mEq/L where the sensitivity was 54.4% and specificity was 54.3%.

### Survival differences for serum bicarbonate groups

**Fig 3:**
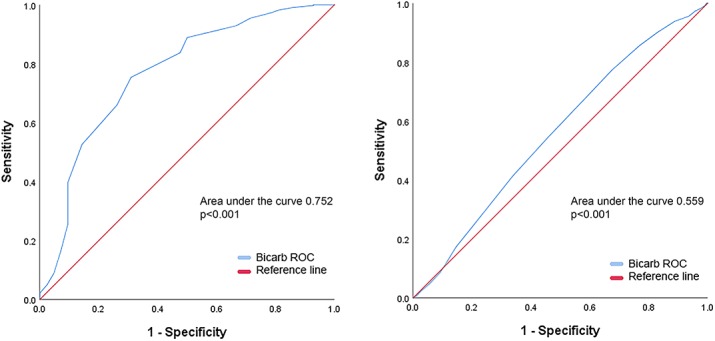
Kaplan Meier curves of the relationship between overall survival and serum bicarbonate levels (Left) Kaplan Meier curve of 30-day mortality in patients with normal, low and high serum bicarbonate (Right) Kaplan Meier curve of 5-year overall survival in patients with normal, low and high serum bicarbonate.

To assess the utility of bicarbonate further we examined the 30-day and 5-year survival of patients using Kaplan-Meier curves (Table 4, Fig 3). There was a significant difference between bicarbonate groups at 30-days (p<0.001) and 5 years (p<0.001) (Table 4). The low bicarbonate group had the poorest 30-day overall survival (OS) of 91.6% (95% CI 88.3–95.1%). In pairwise comparison, low bicarbonate had a significantly lower 30-day OS than normal bicarbonate at 98.8% (p<0.001, 95% CI 98.3–99.4%) and high bicarbonate at 99.2% (p<0.001, 95% CI 98.2–100.0%) (Table 4, Fig 3). Comparatively, there was no significant difference in OS between normal and high bicarbonate (p = 0.6) (Table 4, Fig 3). Similarly, for 5-year OS low bicarbonate had the lowest OS of 48.2% (95% CI 41.5–55.9%). In pairwise comparison, this was significantly lower than normal bicarbonate 5-year OS of 63.0% (p<0.001, 95% CI 60.1–66.0%) and high bicarbonate 5-year OS of 65.4% (p<0.001, 95% CI 59.5–71.9%) (Table 4, Fig 3). There was no statistical difference in 5-year OS between normal and high bicarbonate (p = 0.3) (Table 4, Fig 3).

**Table 4:** 30 day and 5 year survival differences in patients with low, normal and high serum bicarbonate.

Bicarbonate group	No., (%),	30 day survival (%, 95% CI)	Overall P	Pairwise P	5 year survival (%, 95% CI)	P	Pairwise P
Low (< = 22 mEq/L)	264 (11.9)	91.6 (88.3–95.1)	<0.001	<0.001	48.2 (41.5–55.9)	<0.001	<0.001
Normal (23–29 mEq/L)	1595 (71.7)	98.8 (98.3–99.4)		referent	63.0 (60.1–66.0)		referent
High (> = 30 mEq/L)	364 (16.4)	99.2 (98.2–100.0)		0.3	65.4 (59.5–71.9)		0.6

## Discussion

The current study successfully investigated the peri-operative and long-term prognostic utility of bicarbonate in colorectal cancer patients undergoing resection. Furthermore, it also successfully investigated the association of serum bicarbonate with tumor stage, grade and known highly prognostic markers of systemic inflammation.

The first important novel finding of the current study was that low serum bicarbonate was a significant marker of increased peri-operative mortality. To our knowledge, this is the first time that the utility of serum bicarbonate in the peri-operative setting of cancer has been demonstrated. Prior to this study, a number of general operative scores such as the P-POSSUM score have utilized and incorporated a number of other routine serum tests together to provide an estimation of operative risk [15, 16]. However, there is no prior publication or score that had assessed or incorporated serum bicarbonate level. In the current study we assessed the utility of serum bicarbonate whilst controlling for other significant factors previously utilized in the different POSSUM scores. We chose to specifically utilize factors from the POSSUM scores as opposed to other peri-operative instruments because the POSSUM family of scores has been validated numerous times clinically [16, 17]. In the current study, serum bicarbonate was found to be the most significant factor for peri-operative mortality in multivariable analysis even when adjusted for other key factors such as poor renal function or electrolyte disturbance. Indeed, patients with a low serum bicarbonate had an 8% mortality rate compared to a 1% mortality rate in patients with either a normal or high serum bicarbonate at 30 days post-op. We hypothesize that a low serum bicarbonate is reflective of multifactorial processes, both acute and chronic, which contribute to mortality. In part it reflects the underlying aggressiveness of cancer and this is confirmed by the association of low serum bicarbonate with T stage, markers of systemic inflammation and tumor grade. It also reflects critically unwell patients with metabolic acidosis and patients with underlying co-morbidities such as poor baseline renal function. The current study unfortunately did not have data identifying the critically unwell patients or on surrogates such as lactate levels, however these were operable colorectal patients and were well enough to undergo operation and thus it is unlikely that there were large numbers of patients with extreme acid base disturbance. Whilst the current study recognizes that clinicians can identify unwell patients with significant acid-base disturbances who are likely to die, not all patients with milder degrees of acid-base abnormality present as unwell. Conversely, not all patients who appear unwell will have acid-base lability. This study instead suggests that serum bicarbonate provides a reasonable objective surrogate that reduces intra-clinician variability in terms of mortality prediction. Overall, these findings support the use of serum bicarbonate as an adjunct factor to predict peri-operative mortality in the setting of colorectal cancer patients undergoing resection of their primary. Future work should incorporate it into peri-operative mortality tools such as the P-POSSUM score.

The second central finding of the current study was that low serum bicarbonate was not associated with long term survival in colorectal cancer. This is also the first time, to our knowledge, that the long-term prognostic utility of bicarbonate has been reported for colorectal cancer in the literature. In the current study, serum bicarbonate was highly significant in univariable analysis. Furthermore, there was a substantial divergence in survival beyond the peri-operative period out to five years in the low serum bicarbonate group compared to the normal/high bicarbonate groups. However, after accounting for other key factors such as markers of systemic inflammation, serum bicarbonate was not by itself prognostic. This suggests that these other factors are more important to the underlying biology for long term survival than serum bicarbonate, irrespective of whether it is cancer specific or overall survival. We believe that this novel finding is of particular importance contextually as there has been considerable recent interest in altering pH as a therapeutic target in cancer.

The concept of manipulating pH as a therapeutic strategy in cancer has been predicated on past literature demonstrating a link between low tumoral pH, cancer related inflammation and pro-tumorigenic activity [5]. Low tumoral pH is a key characteristic of the tumor microenvironment that can modulate the delicate balance of pro and anti-tumorigenic inflammatory players. Recent literature suggests that low pH can shift activity towards pro-tumorigenic activity by inhibiting tumor infiltrating lymphocytes and by polarizing tumor associated macrophages towards the M2 phenotype [6]. If this tumoral pH could be elevated, then theoretically inflammatory pro-tumorigenic processes could be ameliorated. This theory has been clinically proven in mouse models by Robey *et al*. (2009) who demonstrated that oral serum bicarbonate supplementation can raise tumoral pH and inhibit the rate of spontaneous metastasis [8]. However, the literature also suggests that bicarbonate supplementation at doses required to raise human pH in humans would not be tolerated [18]. Whilst the current literature provides several theoretical mechanisms for manipulating tumoral pH, to our knowledge no therapeutic intervention has thus far been found to be effective [7]. Despite this, the wider public have increasingly utilized and propagated numerous interventions aimed at increasing either serum or tumoral pH. A recent systematic review found that these therapies, which include alkaline diets, alkaline water and bicarbonate supplementation, have no good evidence to support their use as effective anti-cancer treatments [9]. The current study’s results did provide some rationale for targeting pH. The study did identify a novel finding that low serum bicarbonate was significantly associated with elevated markers of systemic inflammation. Low LMR and high NLR, biomarkers widely studied and previously found to be associated with poor OS and CSS in CRC, were significantly associated with low serum bicarbonate in the current study [14, 19]. However, serum bicarbonate by itself was not significantly associated with OS when accounting for these factors. This suggests that manipulation of serum bicarbonate levels by itself should not confer any survival benefit. These results certainly provide additional insight into the ongoing debate regarding bicarbonate supplementation as a therapeutic strategy. The results also suggest that future work in this area should be more focused on targeting tumoral pH directly rather than raising tumoral pH through serum bicarbonate. Proposed targets in this setting include ion channels involved in proton transport such as NHE-1 or enzymes such as Carbonic anhydrase IX (CAIX) which catalyze hydration of carbon dioxide [7, 20].

The strength of the current study was that it was able to succesfully investigate its stated aims in a large consecutive cohort of surgical patients with long term follow up. Moreover, it provided multiple novel findings relating to the prognostic utility of bicarbonate and was able to demonstrate an important link between systemic inflammation and serum bicarbonate. There were however limitations to the current study. Firstly, we lacked information on whether the nature of a patient’s presentation was elective or emergent. However even in prior scoring systems for peri-operative mortality, emergent presentations were only one prognostic factor amongst many [15, 16]. Indeed, when we adjusted for many of these factors, serum bicarbonate remained significantly prognostic. Moreover, there is evidence in the literature to suggest that urgent operations in colorectal cancer when adjusted for other risk factors appropriately is not in itself associated with differences in overall survival or disease free survival [21, 22]. Another limitation was that the current study lacked complete information on co-morbidities or medications that could confound the current results. However, we did attempt to account for some important confounders such as renal function by including factors such as creatinine, urea and electrolytes. It should be noted that in prior validated scores such as the POSSUM family of scores, co-morbidities were grouped into one or two broad variables which contributed only a small component of the predicted peri-operative mortality[16, 17]. This is important as the magnitude of serum bicarbonate on peri-operative mortality appears larger than that of co-morbidities observed in these prior scores. For longer term mortality, confounders would be unlikely to change the negative finding that has already been demonstrated. Another potential limitation was that we chose to use categorical variables instead of using continuous data. This was done as the principal purpose of the study was to provide broad novel insight that could easily be understood. In future work where fine tuning a usable clinical model is required, the use of splines for incorporating continuous data should be considered. A final limitation was that the study lacked data on cancer specific survival and patient specific data on chemotherapy and radiotherapy. Future studies should ideally incorporate these data to have a clearer understanding of the role of serum bicarbonate in prognosis. The current study also points towards studying the relationship between tumoral pH and serum bicarbonate. Certainly, a good understanding of the underlying biology dictating tumoral pH will better guide any potential therapeutic interventions.

In conclusion, the current study added to the literature significantly by assessing the peri-operative and long-term utility of serum bicarbonate. Furthermore, it shed further insight into serum bicarbonate as a potential therapeutic agent for cancer patients.

## Supporting information

S1 Table REMARK checklist for the current study. (DOCX)
