# Correction: Serum bicarbonate is a marker of peri-operative mortality but is not associated with long term survival in colorectal cancer

**DOI:** 10.1371/journal.pone.0244898

**Published:** 2021-08-05

**Authors:** Joseph Chung Yan Chan, Connie Irene Diakos, Alexander Engel, David Lok Hang Chan, Nick Pavlakis, Anthony Gill, Stephen John Clarke

There is an error in Table 2. In the second column of Table 2, the numbers of patients for the ‘low’ and ‘normal’ groups have been switched in the rows under the variable named ‘Creatinine,’. Currently there are 1791 patients with ‘low’ creatinine and 86 patients with ‘normal’ creatinine. There should instead be 86 patients with ‘low’ creatinine and 1791 patients with ‘normal’ creatinine. This corresponds with the number originally referred to in Table 1. The hazard ratios and p values remain unchanged in their assigned columns and rows. The footnote in Table 2 relating to the variable ‘creatinine’ has also been altered, to bring additional clarity to the values for male and female patients.

**Table 2:** Primary univariate and multivariate analysis of key serum, inflammatory and genomic markers in relation to 30-day overall survival in CRC patients undergoing resection of their primary tumor.

Clinicopathologic variables	No., (%),	Univariate analysis, HR (95% CI)	P	Multivariate analysis, HR (95% CI)	P
**Gender**					
M	1104 (49.7)	1 (referent)	0.783		
F	1119 (50.3)	1.089 (0.594–1.995)			
**Age**					
≤70	887 (39.9)	1 (referent)	<0.001	1 (referent)	0.004
>70	1336 (60.1)	13.49 (3.260–55.81)		8.497 (2.020–35.75)	
**T stage**					
1	140 (6.3)	1 (referent)	0.075		0.375
2	349 (15.7)	0.199 (0.018–2.199)			
3	1174 (52.8)	1.320 (0.310–5.613)			
4	560 (25.2)	2.141 (0.495–9.265)			
**N stage**					
0	1193 (53.7)	1 (referent)	0.923		
1	657 (29.6)	0.980 (0.485–1.981)			
2	373 (16.8)	1.161 (0.517–2.607)			
**M stage**					
0	2123 (95.5)	1 (referent)	0.398		
1	100 (4.5)	1.659 (0.513–5.370)			
**Site**					0.714
Right-sided colon	1043 (46.9)	1 (referent)	0.113		
Left-sided colon	611 (27.5)	0.648 (0.313–1.345)			
Rectum	569 (25.6)	0.413 (0.170–1.004)			
Grade*					
Low	919 (45.8)	1 (referent)	0.009	1 (referent)	0.043
Mod	698 (34.8)	2.841 (1.226–6.583)		2.218 (0.923–5.329)	
High	389 (19.4)	3.878 (1.607–9.356)		3.153 (1.282–7.757)	
**MMR-BRAF status** †					
MMRp/BRAFV600E	175 (9.3)	1 (referent)	0.671		
MMRd/BRAFwt	96 (5.1)	0.731 (0.142–3.765)			
MMRd/BRAFV600E	215 (11.5)	0.962 (0.294–3.152)			
MMRp/BRAFwt	1388 (74.1)	0.624 (0.239–1.631)			
**Creatinine** (mmol/L) ‡					
Low < 45/60	86 (3.9)	1.714 (0.405–7.254)	0.001	1.417 (0.325–6.188)	0.036
Normal 45–90 / 60–110	1791 (80.6)	1 (referent)		1 (referent)	
High >90 / 110	346 (15.5)	3.516 (1.868–6.620)		2.464 (1.242–4.889)	
**WBC (x10**^**9**^ **cells /L)**					
4–10	1697 (76.3)	1 (referent)	0.002		0.383
10.1–20 or 3.1–4	484 (21.8)	1.417 (0.703–2.856)			
>20 or < 3	42 (1.9)	6.697 (2.343–19.142)			
**Lymphocyte to monocyte ratio (LMR)**					
Low (<2.38)	1148 (51.6)	1 (referent)	0.298		
High (> = 2.38)	1075 (48.4)	0.721 (0.389–1.335)			
**Neutrophil to lymphocyte ratio (NLR)**					
Low (<3.75)	1143 (51.4)	1 (referent)	0.040		0.964
High (> = 3.75)	1080 (48.6)	1.936 (1.030–3.639)			
**Haemoglobin (mg/dL)**					
13-16g/ dl	783 (35.2)	1 (referent)	0.111		0.222
11.5–12.9 or 16.1–17	576 (25.9)	2.739 (1.105–6.786)			
10–11.4 or 17.1–18	528 (23.8)	2.825 (1.127–7.081)			
<10 or > 18	336 (15.1)	2.684 (0.973–7.401)			
**Urea** (mg/dL)					
<7.6	1823 (82.1)	1 (referent)	<0.001		0.338
7.6–10.0	244 (10.9)	1.423 (0.547–3.707)			
10.1–15	125 (5.6)	4.092 (1.776–9.428)			
>15	31 (1.4)	9.847 (3.436–28.22)			
**Sodium** (mEq/L)					
>135	1937 (87.1)	1 (referent)	0.006	1 (referent)	0.036
131–135	248 (11.2)	2.053 (0.944–4.465)		2.042 (0.920–4.534)	
126–130	34 (1.5)	3.753 (0.898–15.68)		1.731 (0.405–7.394)	
<126	4 (0.2)	15.94 (2.175–116.8)		12.83 (1.654–99.52)	
**Potassium (mEq/L)**					
3.5–5.0	2045 (92.0)	1 (referent)	0.397		
3.2–3.4 or 5.1.-5.3	136 (6.1)	1.670 (0.595–4.693)			
2.9–3.1 or 5.4–5.9	40 (1.8)	2.945 (0.709–12.231)			
<2.9 or >5.9	2 (0.1)	0.01 (na)			
**Bicarbonate** (mEq/L)				5.983 (3.006–11.906)	<0.001
Low (< = 22)	1595 (71.7)	7.535 (4.015–14.14)	<0.001	1 (referent)	
Normal (23–29)	264 (11.9)	1 (referent)		1.192 (0.341–4.163)	
High (> = 30)	365 (16.4)	0.719 (0.212–2.439)		1.192 (0.341–4.163)	

* 133 patients missing tumor grade,

^†^349 patients missing MMR-BRAF status,

^‡^ Lower normal cut point is 45 mmol/L for females and 60 for males; upper normal cut point is 90 mmol/L for females and 110 for males.

There is also an error in Table 3. In the second column of Table 3, in the rows under the variable named ‘Creatinine,’, the numbers of patients for the ‘low’ and ‘normal’ groups have been switched. Currently there are 1791 patients with ‘low’ creatinine and 86 patients with ‘normal’ creatinine. There should instead be 86 patients with ‘low’ creatinine and 1791 patients with ‘normal’ creatinine. This corresponds with the number originally referred to in table 1. The hazard ratios and p values remain unchanged in their assigned columns and rows.

**Table 3:** Primary univariate and multivariate analysis of key serum, inflammatory and genomic markers in relation to 5-year overall survival in CRC patients undergoing resection of their primary tumor.

Clinicopathologic variables	No., (%),	Univariate analysis, HR (95% CI)	P	Multivariate analysis, HR (95% CI)	P
**Gender**					
M	1104 (49.7)	1 (referent)	0.013		0.077
F	1119 (50.3)	0.819 (0.700–0.959)			
**Age**					
≤70	887 (39.9)	1 (referent)	<0.001	1 (referent)	<0.001
>70	1336 (60.1)	1.805 (1.521–2.142)		1.633 (1.334–2.000)	
**T stage**					
1	140 (6.3)	1 (referent)	<0.001	1 (referent)	<0.001
2	349 (15.7)	0.719 (0.419–1.232)		0.660 (0.355–1.229)	
3	1174 (52.8)	1.988 (1.265–3.126)		1.402 (0.825–2.381)	
4	560 (25.2)	5.383 (3.413–8.491)		2.835 (1.647–4.882)	
**N stage**					
0	1193 (53.7)	1 (referent)	<0.001	1 (referent)	<0.001
1	657 (29.6)	1.798 (1.491–2.168)		1.281 (1.025–1.601)	
2	373 (16.8)	3.728 (3.067–4.532)		2.835 (1.647–4.882)	
**M stage**					
0	2123 (95.5)	1 (referent)	<0.001	1 (referent)	<0.001
1	100 (4.5)	4.221 (3.234–5.509)		2.478 (1.771–3.468)	
**Site**					
Right-sided colon	1043 (46.9)	1 (referent)			
Left-sided colon	611 (27.5)	1.025 (0.851–1.234)	0.218		
Rectum	569 (25.6)	0.859 (0.706–1.046)			
Grade*					
Low	919 (45.8)	1 (referent)	<0.001	1 (referent)	<0.001
Mod	698 (34.8)	1.306 (1.073–1.589)		1.381 (1.106–1.724)	
High	389 (19.4)	2.037 (1.640–2.529)		1.621 (1.248–2.105)	
**MMR-BRAF status** †					
MMRp/BRAFV600E	175 (9.3)	1 (referent)	<0.001	1 (referent)	0.010
MMRd/BRAFwt	96 (5.1)	0.432 (0.270–0.691)		0.778 (0.470–1.286)	
MMRd/BRAFV600E	215 (11.5)	0.433 (0.304–0.618)		0.501 (0.335–0.749)	
MMRp/BRAFwt	1388 (74.1)	0.545 (0.424–0.701)		0.786 (0.592–1.043)	
**Lymphocyte to monocyte ratio (LMR)**					
Low (<2.38)	1148 (51.6)	1 (referent)	<0.001	1 (referent)	<0.001
High (> = 2.38)	1075 (48.4)	0.490 (0.416–0.577)		0.637 (0.524–0.776)	
**Neutrophil to lymphocyte ratio (NLR)**					
Low (<3.75)	1143 (51.4)	1 (referent)	<0.001		0.422
High (> = 3.75)	1080 (48.6)	1.918 (1.634–2.251)			
**Bicarbonate** (mEq/L)					
Low (< = 22)	264 (11.9)	1.912 (1.545–2.365)	<0.001	1.277 (0.996–1.638)	0.075
Normal (23–29)	1595 (71.7)	1 (referent)		1 (referent)	
High (> = 30)	364 (16.4)	0.885 (0.705–1.111)		1.221 (0.934–1.597)	
**Creatinine** (mmol/L) ‡					
Low < 45/60	86 (3.9)	2.061 (1.454–2.922)	<0.001	1.990 (1.320–3.001)	0.002
Normal 45–90 / 60–110	1791 (80.6)	1 (referent)		1 (referent)	
High >90 / 110	346 (15.5)	1.730 (1.423–2.104)		1.286 (0.967–1.710)	
**Urea** (mg/dL)					
<7.6	1823 (82.1)	1 (referent)	<0.001	1 (referent)	0.002
7.6–10.0	244 (10.9)	1.329 (1.047–1.686)		1.074 (0.783–1.472)	
10.1–15	125 (5.6)	1.980 (1.490–2.630)		1.601 (1.110–2.307)	
>15	31 (1.4)	3.209 (1.949–5.284)		2.785 (1.520–5.101)	

* 133 patients missing tumor grade,

^†^349 patients missing MMR-BRAF status,

^‡^Lower normal cut point is 45 mmol/L for females and 60 for males; upper normal cut point is 90 mmol/L for females and 110 for males.

In Tables 2 and 3, the footnotes relating to the variable ‘creatinine’ have been altered to bring additional clarity to the values for male and female patients.
